# Boosting weight loss after conversional Roux-en-Y Gastric Bypass with liraglutide and placebo use. A double-blind-randomized controlled trial

**DOI:** 10.1097/JS9.0000000000000990

**Published:** 2023-12-14

**Authors:** Mohamed Hany, Bart Torensma, Mohamed Ibrahim, Ahmed Zidan, Ann S.S. Agayby, Mohamed H. Abdelkhalek, Iman El Sayed

**Affiliations:** a Department of Surgery; b Biomedical Informatics and Medical Statistics Department, Medical Research Institute; c Madina Women’s Hospital; d Department of Pharmacology, Alexandria University, Egypt; e Clinical Epidemiologist, Leiden University Medical Center (LUMC), Leiden, The Netherlands

**Keywords:** glucagon-like peptide-1 (GLP-1) analog, gut hormones incretins, metabolic biomarkers, revisional one-anastomosis gastric bypass, weight loss liraglutide

## Abstract

**Background:**

Conversional bariatric surgery inherently has less weight loss (WL) compared to primary procedures. Adjunctive use of the GLP-1 analog, liraglutide with conversional Roux-en-Y Gastric Bypass (cRYGB) may maximize the WL benefits of surgery.

**Material and methods:**

This single-center randomized double-blind placebo-controlled trial included 80 patients randomized into two groups; the liraglutide group (40 patients) who received daily injections of liraglutide, and the placebo group (40 patients) who received normal saline starting at 6 weeks from cRYGB and continued for 6 months. After discontinuing the drugs at 6 months and unblinding, the patient were followed up to 12 months. The endpoints were percentage of total weight loss (%TWL) and percentage of excess weight loss (%EWL), and changes in the metabolic biomarkers, and complications within 30 and 90 days according to the global outcome benchmark (GOB) stratification.

**Results:**

In total, 38 patients in the liraglutide group and 31 in the placebo group completed the 24 weeks. Liraglutide group experienced better WL with a significantly higher mean %TWL at 1 month (10.27±1.39 vs. 8.41±2.08), at 6 weeks (12.65±1.77 vs. 10.47±2.23), at 6 months (18.29 ±1.74 vs. 15.58 ±1.65), and at 12 months 24.15±2.35 versus 22.70±2.13 (all *P*<0.001). For %EWL, this was also significantly higher in the liraglutide group at all time points. A %TWL of greater than 20% at 6 months of treatment was recorded in six (15.8%) patients in the liraglutide group and none in the placebo group (*P*=0.029). Both groups had comparable changes in metabolic biomarkers. Adverse events were recorded in 11 (27.5%) patients in the liraglutide, with no adverse events in the placebo group (*P*<0.001). Both groups had Clavien–Dindo scores I and II (5.0 and 2.5%), and GOB values indicated that 90.0 and 97.5% were low-risk patients.

**Conclusion:**

Adjunctive use of liraglutide with cRYGB gives significantly higher WL and resolution of associated medical problems.

## Introduction

Highlights

- The liraglutide group experienced better weight loss with a significantly higher mean percentage of total weight loss, and percentage of excess weight loss at 4, 6 weeks, 6 months, and 12 months.
- Both groups had comparable changes in metabolic biomarkers.
- Adverse events were significantly more recorded in the liraglutide group (27.5%) compared to the placebo group (0.0%).

Laparoscopic sleeve gastrectomy (LSG) has gained much popularity, constituting around 55% of worldwide bariatric procedures in 2018^1^. LSG has high reported revision rates, ranging from 10 to 22% according to a systematic review^2^, whereby weight loss failure in the form of weight regain (WR) and insufficient weight loss (IWL) accounts for about 70% of revisions after LSG^1^. Roux-en-RYGB gastric bypass (RYGB) is still the most popular as a revisional procedure for failed LSG with good outcomes in terms of weight loss and resolution of associated medical problems^1,3^. Nevertheless, revisional/conversional bariatric metabolic surgery (BMS) inherently has worse outcomes than primary BMS^4,5^.

Liraglutide is an analog of the gut-derived incretin glucagon-like peptide-1 (GLP-1) with a long half-life of about 13 h that was used initially to treat type 2 diabetes mellitus^6,7^. The glycemic control benefits are shown at a dose of 1.8 mg/day, while the weight loss effect is shown at a dose of 3.0 mg/day, with a reported total body weight loss (%TWL) of about 8%^7^. This dose-dependent weight reduction effect is mediated by gastrointestinal and central mechanisms, including delayed gastric emptying and reduced meal frequency and size through appetite regulation^6,7^. Data showing significant augmentation of weight loss with liraglutide after different bariatric procedures, including only one randomized controlled trial evaluating liraglutide’s extra weight loss benefit after primary LSG^6,8–13^. Unknowns are the effects after conversional surgery. Therefore, this study was designed as a double-blind, randomized, placebo-controlled trial aiming to assess the impact of liraglutide administration after conversional RYGB for failed LSG (cRYGB) on weight loss, resolution of associated medical problems, and gut hormone changes.

## Materials and methods

### Study design

This was a single-center randomized, double-blind, placebo-controlled trial that aimed to assess the additional weight reduction effect of liraglutide after cRYGB compared to the cRYGB with placebo use. Patients who had cRYGB between March 2022 and September 2022 at one specialized bariatric center were offered to participate in the study. The institute covered the cost of liraglutide/placebo treatment. The Ethics Committee approved the study protocol and registered under the institutional registration number: IORG0008812. It was also registered at clinical trials.gov under the NCT number: NCT05285397. The CONSORT checklist was used (Appendix 1)^14^ (Supplemental Digital Content 1, http://links.lww.com/JS9/B571) (Supplemental Digital Content 2, http://links.lww.com/JS9/B572). All participants signed informed consents for the operative procedure and for participating in the study.

### Study endpoints

*Primary endpoint* was weight loss measured by the %TWL and excess weight loss (%EWL) measured throughout the liraglutide/placebo treatment at 1 month, 6 weeks, and 6 months. After discontinuing the drugs at 6 months and unblinding the patient, EWL and TWL were recorded at 12 months.

*Secondary endpoints* were changes in metabolic biomarkers measured at the end of treatment, including fasting glucose levels, HbA1c, HOMA-IR, fasting insulin level, GLP-1 gastric inhibitory peptide (GIP), leptin, ghrelin, and peptide-YY (PYY) secreted from ileum and colon.

### Inclusion and exclusion criteria

*Inclusion criteria* for enrollment in the study were patients’ age between 18 and 60 years, and IWL or WR after previous LSG as indications for cRYGB. IWL was defined as a %EWL of less than 50% through 18 months after LSG, WR was defined as a reported weight increase after achieving greater than 50% of %EWL^15^.

*Exclusion Criteria* included the history of previous bariatric surgery or any gastric surgery before LSG, major complications after the cRYGB as a leak, previous use of liraglutide, previous attacks of pancreatitis, and any contraindications to liraglutide use as a family or personal history of malignancies as multiple endocrine neoplasia or medullary thyroid cancer^16^.

#### Liraglutide/placebo pens

Patients in the liraglutide group received daily subcutaneous injections of liraglutide (Saxenda) at increasing doses starting on postoperative week-6 with 0.6 mg daily injections, increasing by 0.6 mg every week to reach the maximum dose of 3 mg daily after 5 weeks, the liraglutide therapy continued for 24 weeks. The patients in the placebo group received pens filled with normal saline (NaCl 0.9%) in the same increasing dose regime. Both patients and the medical staff directly supervising the patients were blinded. Used liraglutide pens were renewed with new ones to confirm compliance with the treatment regimen and distributed with random numbers logged in a database by two unblinded investigators.

### Preoperative and postoperative care

In the outpatient clinic, a multidisciplinary team assessed all participants, including a bariatric surgeon, a nutritionist, a psychiatrist, and an endocrinologist. All participants were instructed to hypocaloric diet with a 500 kcal energy deficit. The 6-week interval between surgery and the start of liraglutide/placebo treatment is to allows for the resolution of the surgery-induced gastro-intestinal upsets and the restoration of good oral intake before starting the liraglutide treatment. The complete workup is presented in Appendix 2 (Supplemental Digital Content 3, http://links.lww.com/JS9/B573).

### Measurement of metabolic and hormonal biomarkers

Peripheral blood samples were collected and allowed to clot at room temperature for 30 min, followed by centrifugation at 4000 rpm for 10 min at 4°C. Subsequently, the serum was stored at − 80°C for analysis. The complete analysis plan is presented in Appendix 3 (Supplemental Digital Content 4, http://links.lww.com/JS9/B574).

### Data collection

*Preoperative data* about the previous LSG included preoperative weight, nadir weight, and the time lapse between LSG and cRYGB. Preoperative data included patients’ demographics, BMI, associated medical problems, routine preoperative endoscopy and imaging results, and preoperative laboratory tests.

*Postoperative data* included the changes in liraglutide/placebo injections doses, weight loss over time from previous LSG and after cRYGB, reflux symptoms, side effects of liraglutide treatment, and causes of treatment discontinuation. Changes in pancreatic and gut hormones and resolution of associated medical problems were recorded at the end of treatment. Postoperative complications, length of stay, readmission rates, intensive care admissions were scored up to 90 days, according to the Global Outcome Benchmark values^17^ and Clavien–Dindo classification^18^.

### Surgical procedure

The same team performed all the cRYGB procedures, including two main surgeons at one bariatric center. Patients with preoperatively diagnosed hiatal hernia and/or cholecystitis had concomitant crural repair and/or cholecystectomy with the cRYGB procedure. A full description of the technique is provided in Appendix 4 (Supplemental Digital Content 5, http://links.lww.com/JS9/B575).

### Statistical analysis

For the analyses, we used descriptive and inferential statistics. All data were first tested for normality using the Kolmogorov–Smirnov, Q–Q plot, and Levene’s tests. Categorical variables were expressed as *n* (%). Continuous normally distributed variables were expressed with their means and SDs, while non-normally distributed variables were expressed with their medians and interquartile ranges. When appropriate, categorical variables were tested using Pearson’s χ^2^ test or Fisher’s exact test. Continuous normally distributed data were tested with the student’s *t*-test for independent samples. For non-normally distributed data, the Mann–Whitney *U*-test was used for independent samples. A mixed design repeated measures ANOVA test was conducted to study the main effect of postoperative time, the main effect of combined Liraglutide and surgery versus placebo, and if the interaction is present in the form of change pattern of weight, BMI, %TWL, and %EWL along different postoperative periods between both two groups. Absolute benefit increase, relative risk increase, and Number Needed to Treat (NNT) were calculated for greater than 20%TWL outcome. All statistical tests were conducted using IBM SPSS statistics program version 28 (IBM SPSS Statistics for Windows, Version 28.0. IBM Corp.) and R software packages at a ≤0.05 significance level.

### Sample size calculation

The sample size was calculated using G*power Version 3.1.9.5, based on an effect size of 0.667 for TWL% between the liraglutide and placebo groups, with a power of 0.8 and an alpha level of 0.05. This calculation resulted in a minimum required sample size of 37 patients per group. Considering a potential 10% loss to follow-up or dropout and the possibility of no effect in either the liraglutide or placebo group, a total of 81.4 patients were included in the study, rounded up to a final total of 80 (40 in each group).

### Randomization

A double-blind randomization procedure was performed in this study, using the closed envelopes technique by two unblinded investigators. The patients and the staff supervising them where all blinded to the study groups, including the nursing, medical doctors, and bariatric surgeons. Unblinded staff was not involved with any patient care.

### Data capture

The analysis was performed on a blinded dataset after the completion of medical/scientific review. All protocol violations were identified and resolved, and the dataset was declared complete. All data were collected in a data management system (Castor EDC, Amsterdam, The Netherlands; https://data.castoredc.com), handled according to Good Clinical Practice guidelines, Data Protection Directive certificate, and complied with Title 21 CFR Part 11. Furthermore, the data centers, where all the research data were stored, were certified according to ISO27001, ISO9001, and Dutch NEN7510.

### Theory

The literature suggests that a %TWL of less than 20% is observed 1-year after cRYGB^19–21^. Introducing liraglutide may achieve additional weight loss that might not be possible with the surgery alone, especially in conversional BMS, which typically has lower weight loss results than primary BMS. To our knowledge, this is the first double-blind, randomized, placebo-controlled trial comparing the effects of adjunctive liraglutide with placebo in patients undergoing cRYGB over a 12-month follow-up period, aiming to test this hypothesis.

## Results

This double-blind, randomized, placebo-controlled trial included at the start 80 patients, 40 in the liraglutide group and 40 in the placebo group.

### Lost to follow-up

**Figure 1:**
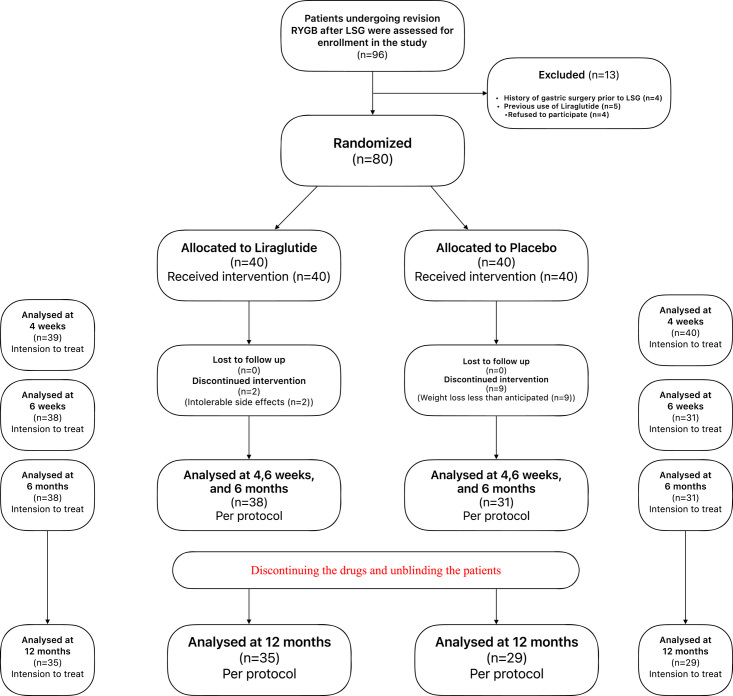
CONSORT flow diagram.

After 6 months,11 patients discontinued the liraglutide/placebo treatment, leaving 38 patients in the liraglutide group and 31 in the placebo group. Two patients discontinued the treatment in the liraglutide group due to severe intolerable side effects. In comparison, nine patients discontinued the treatment in the placebo group because the weight loss results were less than anticipated by them. After discontinuing the drugs, four patients in the liraglutide and two patients in the control group were lost to follow-up. A total of 35 (liraglutide) and 29 (placebo) patients were in the per protocol analyzed at 12 months postoperatively (CONSORT flow diagram, Fig. 1).

### Baseline characteristics

There were no significant differences between both groups. Preoperative demographic characteristics, weight loss parameters after the primary LSG, associated medical problems, preoperative imaging, and endoscopy of the patients (Table 1).

**Table 1:** Preoperative demographics, associated medical conditions, GERD endoscopy, and imaging in liraglutide and placebo groups.

	Liraglutide (*n*=38)	Placebo (*n*=31)	Sig.
Mean (±SD) Age in years	38.21 (±9.06)	37.16 (±12.81)	0.692
Female, no (%):	25 (65.8%)	24 (77.4%)	0.290
Male, no (%):	13 (34.2%)	7 (22.6%)	
Mean (±SD) BMI (kg/m^2^) before primary LSG	46.6 (±8.0)	43.3 (±6.2)	0.061
Mean (±SD) Time between LSG and cRYGB in years	7.00 (±3.15)	7.65 (±3.39)	0.416
Mean (±SD) Nadir BMI (kg/m^2^) after LSG	29.98 (±2.53)	31.19 (±3.21)	0.093
Mean (±SD) BMI before cRYGB	35.85 (±0.93)	36.18 (±0.87)	0.135
Mean %EWL before cRYGB	59.71±22.5	51.61±16.74	0.002^*^
Associated medical conditions, no (%):			
Diabetes mellites	3 (7.9%)	2 (6.5%)	^FE^ *P* 1
Hypertension	5 (13.2%)	0 (0.0%)	^FE^ *P* 0.060
Polycystic ovary	3 (7.9%)	3 (9.7%)	^FE^ *P* 1
Dyslipidemia	17 (44.7%)	12 (38.7%)	^FE^ *P* 0.633
Arthritis	4 (10.5%)	1 (3.2%)	^FE^ *P* 0.370
Obstructive sleep apnea	4 (10.5%)	0 (0.0%)	^FE^ *P* 0.122
Preoperative endoscopy:			
Hiatal hernia	16 (42.1%)	19 (61.3%)	^FE^ *P* 0.113
GERD ‘A’	11 (28.9%)	13 (41.9%)	^FE^ *P* 0.485
GERD ‘B’	6 (15.8%)	5 (16.1%)	
Preoperative imaging:			
Cholecystitis	3 (7.9%)	2 (6.5%)	^FE^ *P* 1

Data are presented as mean±SD or frequency(%) as appropriate.

*Significant results ≤0.05.

FE *P*: Fischer Exact significance.

GERD was diagnosed by preoperative routine endoscopy in 17 (44.7%) and 18 (53%) patients in the liraglutide and placebo groups, respectively (*P*=0.270). Grade ‘A’ and grade ‘B’ formed 64.7 and 35.3% in the liraglutide group and 72.2 and 27.7% in the placebo group (*P*=0.485) (Table 1).

The mean BMI values before LSG were 46.6±8.0, and 43.3±6.2 in the liraglutide and placebo groups, respectively, *P*=0.061. The mean nadir BMI values were 29.98±2.53 and 31.19±3.21 in the liraglutide and placebo groups, respectively (*P*=0.093).

The mean time between the LSG and the cRYGB was 7.00±3.15 years in the liraglutide group and 7.65±3.39 years in the placebo group (*P*=0.416). The mean BMI values before cRYGB were 35.85±0.93, and 36.18±0.87 in the liraglutide and placebo groups, respectively (*P*=0.135).

No significant differences between both groups on preoperative routine lab tests and metabolic biomarkers assay (Table 2).

**Table 2:** Preoperative laboratory tests in liraglutide and placebo groups.

	Liraglutide (*n*=38)	Placebo (*n*=31)	
	Mean (SD)	Mean (SD)	Sig.
Cholesterol (mg/dl)	261.55 (61.95)	232.55 (61.95)	0.630
Triglycerides (mg/dl)	159.71 (61.18)	152.68 (52.86)	0.616
ALT (U/l)	30.09 (10.41)	26.22 (12.94)	0.173
AST (U/l)	21.58 (7.40)	22.22 (7.95)	0.732
Hemoglobin (g/dl)	13.02 (1.70)	13.02 (1.28)	0.998
Platelets (^*^10^9^/l)	240.79 (82.08)	269.35 (90.99)	0.175
WBCs (^*^10^9^/l)	7.25 (2.25)	7.44 (2.02)	0.714
Fasting blood glucose (mg/dl)	102.74 (8.86)	103.5 (14.41)	0.766
Fasting insulin (mcU/ml)	12.08 (13.25)	16.56 (10.84)	0.134
HOMA-IR	2.45 (2.36)	2.22 (1.93)	0.672
HbA1C	5.73 (1.20)	5.68 (0.78)	0.821
PYY (PG/ ml)	126.67 (45.59)	125.07 (49.18)	0.889
GLP-1 (PG/ l)	4.71 (1.4)	4.45 (0.62)	0.348
GIP (PG/ ml)	56.32 (11.61)	56.58 (9.54)	0.919
Leptin (NG/ml)	52.64 (13.54)	51.21 (15.81)	0.687
Ghrelin (PG/ ml)	325.05 (97.80)	304.97 (74.98)	0.351
TSH (mIU/l)	2.77 (1.15)	3.10 (1.15)	0.231
FT3 (PG/ ml)	2.72 (0.94)	3.07 (0.91)	0.126
FT4 (NG/dl)	1.27 (0.47)	1.21 (0.54)	0.631

Data are presented as mean±SD.

* Significant results ≤0.05

GIP, gastric inhibitory peptide; GLP-1, glucagon-like peptide-1; HOMA-IR, homeostatic model assessment for insulin resistance; PYY, peptide-YY.

#### Weight loss measured from conversional RYGB and from primary LSG (per protocol analysis)

**Figure 2:**
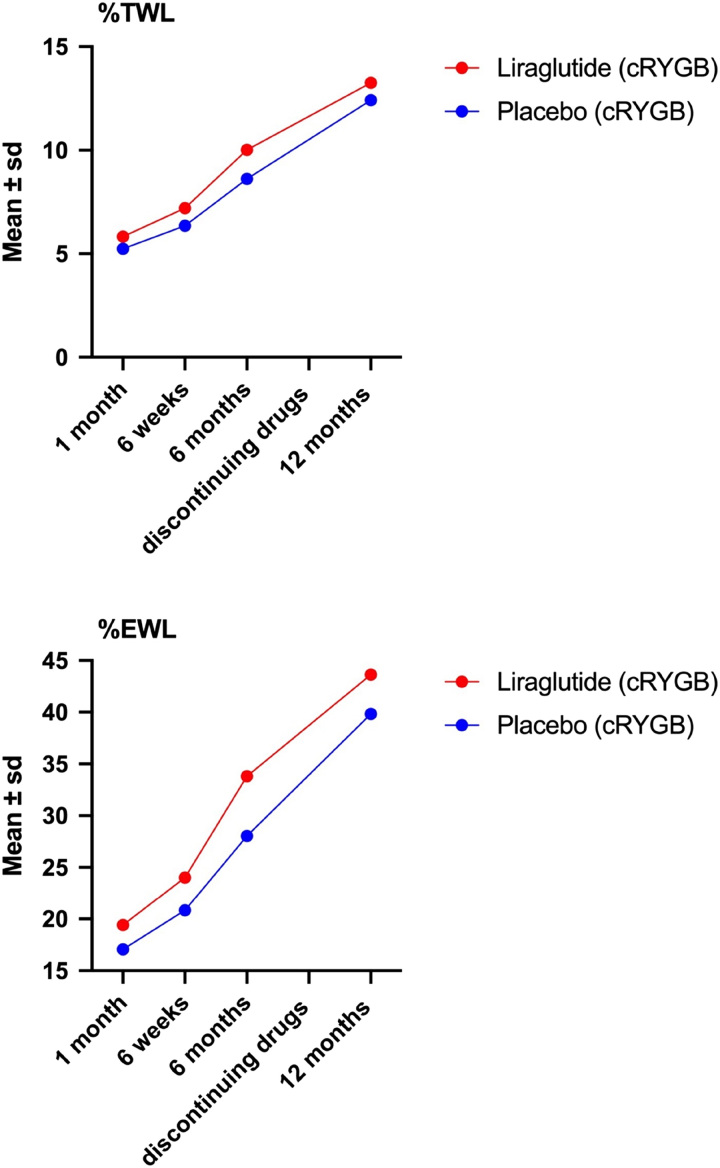
Error bar graph displaying results of mixed design repeated measures analysis of variance (ANOVA) test on average BMI, average TWL% and average %EWL with respective 95% CI separately for patients in the liraglutide and placebo cohorts.

After 1 month of the liraglutide/placebo treatment, the mean BMI was 32.21±0.93 in the liraglutide group and 32.80±0.76 in the placebo group. After 6 weeks, 31.36±0.99 versus 32.10±0.83, after 6 months, 29.38±1.20 versus 30.56±0.84 (*P*<0.001). After discontinuing the drugs at 6 months, the results showed at 12 months 27.22±0.90 versus 27.95±0.75, what was not significant between the groups (*P*=0.912) (Table 3, Fig. 2).

**Table 3:** Mixed design repeated measures analysis of variance (ANOVA) test comparison of BMI, %TWL and % EWL at different follow-up periods between liraglutide and placebo groups up to 12 months.

	Liraglutide (*n*=35) PP	(*n*=40) ITT	Placebo (*n*=29) PP	(*n*=40) ITT	Sig. PP	ITT
BMI:						
BMI at 1 month	32.21^*^±0.93	32.45±1.81	32.79^*^±0.78	33.92±2.74	*P*<0.001^*^ ^†^	*P*<0.001^*^ ^†^
BMI at 6 weeks	31.36^†^±1.01	31.36±0.97	32.08^†^±0.86	33.15±2.67	*P*<0.001^*^ ^‡^	*P*<0.001^*^ ^‡^
BMI at 6 months	29.34^‡^±1.07	29.27±1.07	30.53^‡^±0.86	30.56±0.84	*P*<0.001^*^ ^¶^	*P*<0.001^*^ ^¶^
BMI at 12 months	27.22±0.90	27.22±0.90	27.95±0.75	27.95±0.75	*P*<0.001^*^ ^¶^	*P*<0.001^*^ ^¶^
% total weight loss (%TWL) after conversional RYGB						
%TWL at 1 months	10.27±1.39	10.06^*^±1.55	8.41±2.08	9.31^*^±1.28	*P*<0.001^*^ ^†^	*P*<0.001^*^ ^†^
%TWL at 6 weeks	12.65±1.77	12.51^†^±1.77	10.47±2.23	11.25^†^±1.62	*P*<0.001^*^ ^‡^	*P*<0.001^*^ ^‡^
%TWL at 6 months	18.29±1.74	18.35^‡^±1.72	15.58±1.65	15.53^‡^±1.64	*P*<0.001^*^ ^¶^	*P*<0.001^*^ ^¶^
%TWL at 12 months	24.15±2.35	24.15±2.35	22.70±2.13	22.70±2.13	*P*<0.001^*^ ^¶^	*P*<0.001^*^ ^¶^
% total weight loss (%TWL) from primary LSG						
%TWL at 1 months	28.64±11.11	28.65±11.36	23.29±11.06	20.55±10.92	*P*<0.001^*^ ^†^	*P*<0.001^*^ ^†^
%TWL at 6 weeks	30.53±10.81	30.95±10.82	24.93±10.83	22.35±10.63	*P*<0.001^*^ ^‡^	*P*<0.001^*^ ^‡^
%TWL at 6 months	34.98±10.43	35.50±10.53	28.57±10.28	28.07±10.34	*P*<0.001^*^ ^¶^	*P*<0.001^*^ ^¶^
%TWL at 12 months	39.72±9.33	39.72±9.33	34.62±9.35	34.62±9.35	*P*<0.001^*^ ^¶^	*P*<0.001^*^ ^¶^
% excess weight loss (%EWL) after conversional RYGB						
%EWL at 1 months	33.98^*^±4.83	32.23±5.74	30.32^*^±3.80	26.74±7.57	*P*<0.001^*^ ^†^	*P*<0.001^*^ ^†^
%EWL at 6 weeks	41.85^†^±6.17	41.51±6.04	36.63^†^±5.06	33.23±8.09	*P*<0.001^*^ ^‡^	*P*<0.001^*^ ^‡^
%EWL at 6 months	60.59^‡^±7.05	61.00±7.15	50.61^‡^±5.45	50.41±5.37	*P*<0.001^*^ ^¶^	*P*<0.001^*^ ^¶^
%EWL at 12 months	79.77±7.49	79.77±7.49	73±65±6.04	73±65±6.04	*P*<0.001^*^ ^¶^	*P*<0.001^*^ ^¶^
% Excess weight loss (%EWL) from primary LSG						
%EWL at 1 months	61.90^*^±13.63	61.48±14.66	52.75^*^±18.70	47.13±19.86	*P*<0.001^*^ ^†^	*P*<0.001^*^ ^†^
%EWL at 6 weeks	66.46^†^±12.11	66.89±11.95	57.04^†^±16.92	51.75±18.34	*P*<0.001^*^ ^‡^	*P*<0.001^*^ ^‡^
%EWL at 6 months	76.90^‡^±10.15	77.46±10.17	66.45^‡^±13.38	65.76±13.56	*P*<0.001^*^ ^¶^	*P*<0.001^*^ ^¶^
%EWL at 12 months	88.31±6.23	88.31±6.23	82.04±8.31	82.04±8.31	*P*<0.001^*^ ^¶^	*P*<0.001^*^ ^¶^

Different superscripts denote significant pairwise comparison after Bonferroni correction.

* Significant results ≤0.05.

† Mixed design repeated measures ANOVA test to assess the main effect of time on different parameters with significant linear contrast.

‡ main effect of liraglutide.

¶ and interaction to assess the pattern of change of each quantitative variable along time by liraglutide.

ITT, intention to treat; PP, per protocol.

Measuring from cRYGB after 6 months drug use the mean TWL% was 18.29±1.74 versus 15.58±1.65 (liraglutide vs. placebo). After discontinuing the drugs at 6 months, the results showed at 12 months 24.15±2.35 versus 22.70±2.13. Measured from primary LSG this was 34.98±10.43 versus 28.57±10.28 at 6 months and 39.72±9.33 versus 34.62±9.35 at 12 months (all *P*<0.001) (Table 3, Fig. 2).

For mean %EWL this was from cRYGB after 6 months 60.59±7.05 versus 50.61±5.45 and at 12 months 79.77±7.49 versus 73±65±6.04. Measured from primary LSG this was at 6 months 76.90±10.15 versus 66.45±13.38 and after discontinuing the drugs at 6 months, the results showed at 12 months 88.31±6.23 versus 82.04±8.31 (all *P*<0.001) (Table 3, Fig. 2).

Within both cohorts they all showed a significant decrease in BMI, a significant increase in %TWL, and a significant increase in %EWL over the follow-up period. (Table 3, Fig. 2).

### Weight loss (intention to treat analysis)

There was no cross-over between the groups. However, upon analyzing the data for patients who were lost to follow-up, results at all time points revealed also a significant difference between the two groups. The liraglutide group consistently demonstrated a higher %EWL and %TWL up to 6 months of treatment with the drugs in both cRYGB and from primary LSG analysis. Furthermore, after discontinuing the drugs at 6 months, both %TWL and %EWL were significantly higher at 12 months in favor of the liraglutide group (Table 3).

### NNT with liraglutide

A %TWL of greater than 20% at 6 months of treatment could be achieved in six (15.8%) patients in the liraglutide group and none of the placebo group (*P*=0.029), with an absolute benefit increase of 15.8 and 100% relative risk increase. The NNT is 6.32 patients for an additional patient with a %TWL of greater than 20%.

### Postintervention metabolic biomarkers changes

Metabolic biomarkers after 6 months showed comparable changes from baseline in both cohorts regarding lipid profile, fasting blood sugar, HbA1c, fasting insulin levels, leptin, ghrelin, and PYY levels. The level of GLP-1 decreased significantly in the liraglutide cohort (*P*≤0.001), as the GLP-1 level decreased in the liraglutide cohort and increased in the placebo cohort. Moreover, the levels of GIP increased in both cohorts, with a significantly more increase in the liraglutide cohort (*P*=0.014) (Table 4).

**Table 4:** Comparison of laboratory parameters change from baseline at 6 months between the liraglutide and placebo cohorts.

	Liraglutide (*n*=38)	Placebo (*n*=31)		
	Mean (SD)	Mean (SD)	Difference	Sig.
FBG	−15.21 (15.02)	−15.58 (12.90)	0.370 (−6.32 to 7.06)	0.914
HBA1C	−1.01 (0.81)	−0.82 (0.67)	−0.191 (−0.55 to 0.17)	0.297
HOMA-IR	−1.34 (2.29)	−0.62 (1.68)	−0.73 (−1.71 to 0.26)	0.145
Insulin	15.19 (12.62)	12.50 (13.28)	2.69 (−3.55 to 8.93)	0.392
Cholesterol	−108.55 (60.02)	−90.71 (65.86)	−17.84 (−48.13 to 12.45)	0.244
TGs	−67.16 (59.95)	−71.81 (55.05)	4.65 (−23.28 to 32.57)	0.741
PYY	133.71 (106.91)	108.84 (99.34)	24.87 (−25.17to 74.91)	0.325
GLP−1	−3.23 (4.10)	4.90 (2.86)	−8.14 (−9.81 to −6.46)	<0.001^*^
GIP	18.11 (7.95)	14.42 (3.79)	3.69 (0.76 to 6.61)	0.014^*^
Leptin	−28.33 (14.55)	−28.27 (17.52)	−0.06 (−7.76 to 7.65)	0.988
Ghrelin	−94.47 (98.76)	−78.81 (74.76)	−15.67 (−58.57 to 27.24)	0.469
ALT	−6.32 (6.05)	−4.86 (8.30)	−1.46 (−4.91to 1.99)	0.402
AST	−1.76 (3.14)	−1.02 (3.00)	−0.74 (−2.23 to 0.75)	0.324

Data was calculated as change from baseline at 6 months.

* Significant results ≤0.05.

HOMA-IR, homeostatic model assessment for insulin resistance; GLP-1, glucagon-like peptide-1; GIP, gastric inhibitory peptide; PYY, peptide-YY.

### Liraglutide dosage

Severe and intolerable side effects were the cause of discontinuation of treatment in two (5.26%) patients in the liraglutide group after 3 and 4 weeks of treatment.

Thirty-eight patients continued the 24 weeks of treatment, 31 could reach and tolerate the 3 mg/day dose, while seven patients continued lower doses, including two patients on 2.4 mg/day, four on 1.8 mg/day, and one on 1.2 mg/day.

### Adverse events

The recorded adverse events in the liraglutide group included: nausea and vomiting in six (15.8%) patients, chronic fatigue in one (2.6%) patient, bloating in two (5.26%) patients, severe abdominal pain and vomiting in one (2.6%) patient and severe injection site reactions in one (2.6%) patient. There were no recorded adverse events in the placebo group (*P*<0.001).

### Associated medical problems and GERD

A significantly higher incidence of resolution/improvement of associated medical problems in the liraglutide cohort was recorded for diabetes mellitus (*P*<0.001), hypertension (*P*<0.001), dyslipidemia (*P*=0.041), obstructive sleep apnea (*P*<0.001), and arthritis (*P*=0.008).

Resolution of GERD was recorded in 76.5% of GERD cases in the liraglutide group and 83.3% of GERD cases in the placebo group (*P*=0.611). Only four and three patients were diagnosed with residual GERD in liraglutide and placebo groups; all residuals were grade ‘A’ GERD. (Table 5).

**Table 5:** Resolution/improvement of associated medical conditions and GERD in the liraglutide and placebo cohorts.

	Liraglutide (*n*=38)			Placebo (*n*=31)			
	Preoperative incidence^*^	Incidence at 6 months after the intervention^*^	Incidence of resolution/improvement of associated medical problems^†^	Preoperative incidence^*^	Incidence at 6 months after the intervention^*^	Incidence of resolution/improvement of associated medical problems^†^	Sig.
Diabetes mellitus	3 (7.9%)	0	3 (100%)	2 (6.5%)	1 (3.2%)	1 (50%)	<0.001^‡^
Hypertension	5 (13.2%)	0	5 (100%)	0	0	0	<0.001^‡^
Dyslipidemia	23 (60.5%)	4 (10.5%)	19 (82.5%)	10 (32.3%)	0	10 (100%)	0.041^‡^
Arthritis	4 (10.5%)	1 (2.6%)	3 (75%)	1 (3.2%)	0	1 (100%)	0.008^‡^
Obstructive sleep apnea	4 (10.5%)	2 (5.3%)	2 (50%)	0	0	0	<0.001^‡^
GERD	17 (44.7%)	4 (10.5%)	13 (76.5%)	18 (53%)	3 (9.7%)	15 (83.3%)	0.611

* Proportion of positive associated medical conditions out of total number of patients before treatment per each arm and after 6 months follow-up period.

† Proportion of resolved associated medical conditions after 6 months of treatment with liraglutide/placebo.

‡ Significant results ≤0.05.

### Complications and global outcome benchmarks

In the liraglutide group, 90.0% of cases, and in the placebo group, 95.0% of cases, experienced an uneventful postoperative course within 30 days (*P*=0.871). A Clavien–Dindo class I complication occurred in 5.0% of cases (*P*=1.000) within 90 days in both liraglutide and placebo groups. For Class II complications, the rates were 5.0% in the liraglutide and 2.5% in the placebo group (*P*=0.876). There were no instances of CD≥III complications or mortality. Abdominal pain was present in one patient in the liraglutide group (2.5%) (*P*=0.412). The Global Outcome Benchmark (GOB) values in bariatric surgery indicated that 90.0% of the liraglutide and 97.5% of the placebo group were benchmark cases. Whereby the GOB high-risk patients were scored on obstructive sleep apnea syndrome (OSAS) and pulmonary or cardiovascular symptoms (CVS) complications (Table 6).

**Table 6:** Postoperative complications according to the Global Outcome Benchmark values.

	Liraglutide (*n*=40)	Placebo (*n*=40)	Sig.
Length of stay in days median (min-max)	3 (2-4)	3 (2-4)	1.000
Operation time in min (mean±SD)	103.4±19.8	107.5±13.3	0.576
Intraoperative blood transfusion (*n* %)	0 (0.0)	0 (0.0)	1.000
Postoperative blood transfusion (*n* %)	0 (0.0)	0 (0.0)	1.000
Conversion to open surgery (*n* %)	0 (0.0)	0 (0.0)	1.000
Postoperative morbidity and mortality until 30 days (*n* %)			
Uneventful postoperative course	36 (90.0)	38 (95.0)	0.871
Any complication	4 (10.0)	2 (5.0)	0.374
Mortality	0 (0.0)	0 (0.0)	1.000
Persistent vomiting	1 (2.5)	1 (2.5)	0.974
Diarrhea	1 (2.5)	1 (2.5)	0.974
ICU admission^*^	1 (2.5)	1 (2.5)	1.000
ICU admitted length of stay median (min-max)	1 (1-1)	1 (1-1)	1.000
Postoperative complication until 90 days (*n* %)			
Anastomotic leak	0 (0.0)	0 (0.0)	1.000
Anastomotic stenosis	0 (0.0)	0 (0.0)	1.000
Bleeding	0 (0.0)	0 (0.0)	1.000
Small bowel obstruction	0 (0.0)	0 (0.0)	1.000
Marginal Ulcer or GERD	0 (0.0)	0 (0.0)	1.000
Wound infection	0 (0.0)	0 (0.0)	1.000
Pulmonary or CVS complications	2 (5.0)	1 (2.5)	0.876
Urinary tract infection	0 (0.0)	0 (0.0)	1.000
Abdominal pain	1 (2.5)	0 (0.0)	0.412
Clavien–Dindo (CD) class (*n* %)			
Class I	2 (5.0)	2 (5.0)	1.000
Class II	2 (5.0)	1 (2.5)	0.876
Reintervention CD IIIa	0 (0.0)	0 (0.0)	1.000
Reoperation CDIIIb	0 (0.0)	0 (0.0)	1.000
CCI§ (in patients with at least 1 CD grade)	<20.9	<20.9	1.000
Readmission (*n* %)	4 (10.0)	3 (7.5)	0.147
Benchmark yes (*n* %) (Low-risk patient criteria)	36 (90.0)	39 (97.5)	0.374
Benchmark no (*n* %) (High-risk patient criteria)	4 (10.0)^†^	1 (2.5)^‡^	

* One patient for controlling hypertension and one for OSAS.

† Obstructive sleep apnea syndrome (OSAS) and pulmonary or CVS complications.

‡ Pulmonary or CVS complications.

CCI, Comprehensive Complication Index (§0 no complication, 100 is dead, it achieves this by assigning weighted values to all postoperative complications based on the Clavien–Dindo classification).

CVS, cardiovascular symptoms; GERD, gastroesophageal reflux disease.

## Discussion

In this study, both cohorts exhibited a significant reduction in BMI and a notable increase in both %TWL and %EWL (*P*<0.001). By the 6-month mark, the liraglutide cohort manifested a greater mean %TWL and %EWL. After discontinuing the drugs, the liraglutide group performed significantly better in %TWL and %EWL at the 12-month point. Furthermore, the calculated NNT stood at 6.32 to attain an additional patient with a %TWL surpassing 20%. Achieving a %TWL of 20% within 6 months after cRYGB for failed LSG may represent a significant added benefit for the adjunctive use of liraglutide with surgery.

### Liraglutide and effect on weight loss after BMS

Recent research has examined liraglutide’s role in enhancing post-BMS weight loss^10^. Colbourne *et al*. observed significant weight loss in 89.7% of post-BMS liraglutide users, with 22.1% achieving greater than or equal to 10% TWL. They noted higher median %TWL in revisional RYGB (rRYGB) patients (6.50%) compared to primary BMS (pRYGB) patients (4.10%)^8^. Similarly, Elhag and Ansari^12^ reported notable weight loss in both pRYGB and rRYGB groups postliraglutide, with varying %TWL and greater than or equal to 5% TWL achievements at 6 and 12 months.

A recent RCT by Mok *et al*. explored the effectiveness of liraglutide at a dose of 3.0 mg in a cohort of 35 patients compared to a placebo group of equal size, explicitly targeting individuals who demonstrated inadequate weight loss post-RYGB or LSG surgery. The study revealed a notable mean difference in the percentage of body weight change, favoring liraglutide over placebo, measured at −8.03 (95% CI: −10.39 to −5.66; *P*<0.001). This significant finding bolsters the potential role of adjunct liraglutide therapy, administered at 3.0 mg, in managing weight for patients who show limited weight loss and a suboptimal response to GLP-1 following metabolic surgery^22^.

Another double-blind, placebo-controlled study compared liraglutide to placebo at 1.2 mg/day, 1.8 mg/day, 2.4 mg/day, and 3 mg/day doses. Results showed a dose-dependent increase in %TWL for liraglutide users compared to placebo, ranging from about 7% at 1.2 mg/day to 9.4% at 3 mg/day over 20 weeks. Notably, nearly 30% of participants on the 3 mg/day dose achieved more than 10% TWL in this period^7^.

Furthermore, in a 6-month double-blind, placebo RCT, Thakur *et al*. evaluated 12 patients receiving liraglutide and 11 receiving a placebo, beginning 6 weeks after LSG surgery. The study found that the liraglutide group achieved a mean %TWL of 28.2%±5.7, in contrast to 23.2%±6.2 in the placebo group (*P*=0.116). Additionally, the %EWL was 58.7%±14.3 for the liraglutide group compared to 44.5%±8.6 for the placebo group (*P*=0.043). The average dose of liraglutide administered was 1.41±0.49 mg/day^6^.

Overall, in studies where liraglutide was administered further from the initial weight loss phase postbariatric surgery, the mean %TWL observed ranged from 4 to 10%. This aligns with our study and those by Mok and Thakur *et al*., where liraglutide was administered during the weight loss phase following BMS. These latter studies demonstrated more promising results, surpassing the outcomes of surgery or liraglutide treatment alone. In addition to the promising results observed during the initial 6-month postoperative period, our study showed that after discontinuing the drugs and unblinding, the liraglutide group maintained a higher TWL and EWL at 12 months postoperatively. However, further investigation is required to determine the impact of discontinuing or continuing liraglutide on enhancing weight loss in longer follow-ups beyond 12 months. This aspect has been recommended for thorough examination in a subsequent follow-up study.

### Metabolic biomarkers assay

RYGB is acknowledged for its impact on metabolic biomarkers. Specifically, it results in elevated levels of GLP-1, GIP, and PYY while concurrently decreasing ghrelin and leptin concentrations. This modulation culminates in enhanced glucose homeostasis, characterized by improved insulin secretion and diminished insulin resistance^23,24^. After 6 months, alterations in the metabolic biomarkers were comparable between both cohorts. However, endogenous GLP-1 levels rose in the placebo group and declined in the liraglutide group relative to the baseline measurements taken prior to surgery (*P*<0.001).

The mean GIP level experienced a more pronounced increase in the liraglutide cohort compared to the placebo cohort (*P*=0.014). Data from a randomized, double-blind, placebo-controlled, cross-over trial involving 20 patients with type 2 diabetes also demonstrated a significant elevation in GIP levels upon liraglutide administration. Notably, this increase was independent of weight loss and had a significance level of *P*<0.03^25^.

### Adverse events

In the liraglutide cohort, side effects were observed in 27.5% of patients, predominantly manifesting as mild gastrointestinal symptoms, including nausea, vomiting, and bloating. A maximum dosage of 3 mg/day was well-tolerated by 81.5% of the patients, while the remaining 18.5% persisted on a reduced dose. In postbariatric patients treated with liraglutide, mild to moderate gastrointestinal symptoms were reported, affecting between 12 and 50% of patients. Among these, nausea and vomiting were the most prevalent side effects, impacting up to 40% of the patient population^6,8–10,12,13,26^.

### Cost of liraglutide

The cost of the drug is a significant barrier that affects patients’ compliance. Therefore, the studies that provided the drug to the patients, as well as this study, had lower discontinuation rates, commonly less than 10%, related to side effects of the drug^7,26^. On the other hand, when patients bore the cost of the drug, there were higher discontinuation rates, varying between 26 and 60%. The primary reasons for discontinuation were the drug’s high cost or outcomes that fell short of the patient’s expectations^8–10^. In this study, the less-than-anticipated results were the cause of discontinuation in 22.5% of patients in the placebo cohort, while not reported by the liraglutide cohort.

### Associated medical problems and GERD

After 6 months on liraglutide/placebo, all three patients (100%) with type 2 diabetes mellitus in the liraglutide group achieved normoglycemia without the need for additional medications. In contrast, only one patient (50%) in the placebo group failed to achieve normoglycemia and required ongoing oral medications (*P*<0.001). This outcome suggests an added benefit to the adjunctive use of liraglutide, which is already approved for diabetes treatment at doses ranging from 1.2 to 1.8 mg/day. However, it is crucial to note that the resolution of diabetes cannot be conclusively determined while patients remain on liraglutide treatment.

In this study, GERD resolution was observed in 76.5% of cases in the liraglutide group and 83.3% in the placebo group, with no significant difference between the two (*P*=0.611). GERD is notably one of the primary reasons for seeking conversion after LSG. Previous reports have indicated up to a 100% improvement rate following cRYGB and over a 75% resolution rate upon discontinuation of antireflux medications^2,20,27,28^. This GERD resolution benefit of cRYGB is attainable without satisfactory weight loss outcomes^28^.

#### Global outcome benchmark and complications

The GOB values in this study demonstrated that 90.0 and 97.5% of cases met the benchmarks after conversional surgery from LSG to RYGB. As presented in a 2019 study, the GOB revealed that the proportion of benchmark cases within the case mix of participating centers varied from 4 to 69% in primary BMS^29^. Our study reported a 0% incidence of Clavien–Dindo grade ≥3 complications. According to the GOB values, any complications within 30 days should be less than or equal to 11%, for complication grade ≥IIIa must be less than 5%, and signature complications like staple line leak, dysphagia/stenosis of the gastric tube, postoperative bleeding, small bowel obstruction, and wound infection should be below 0.3–2.2% in primary BMS. In the updated 2021 GOB study for elective secondary BMS^17^, Benchmark cutoffs for conversional BMS were less than 4.5% reintervention less than 8.3% reoperation 90 days postoperatively. No adjusted references were presented for complication ratios. This makes it unclear whether higher complication rates are acceptable. Nevertheless, our study was within the limits of the percentages previously and current GOB. However, this study cohort included only 80 cases, potentially leading to underpowering and underestimation of the GOB. Finally, differences between complications and adverse events, such as gastrointestinal (GIT) symptoms, nausea, vomiting, diarrhea, and abdominal pain, were investigated. Medical examinations differentiated these to ascertain whether they were related to surgical procedures or liraglutide-induced symptoms. This approach provided a better understanding of all the relationships and the side effects of liraglutide use in the postoperative phase.

### Limitations

The study’s main limitation is the follow-up duration; a longer follow-up of more than 1-year is needed to assess the durability of the weight loss outcomes after discontinuing the drugs. Moreover, the resolution of diabetes mellitus while the patients are still on liraglutide treatment is questionable, as the resolution of diabetes should be confirmed when the patients are no longer on medications.

## Conclusion

Adjunctive use of liraglutide at a dose of 3 mg/day with RRAGB has significantly added weight loss and resolution of diabetes mellitus and other associated medical problems to the surgery with good tolerability, even after discontinuing the drugs. The added value from liraglutide use can help overcome the inherently lower weight loss outcomes of cRYGB.

## Ethical approval

The study protocol was approved by the Medical Research Institute of Alexandria University Ethics Committee and was registered under the institutional registration number: IORG0008812.

## Consent

Written informed consent was obtained from the patient for publication and any accompanying images. A copy of the written consent is available for review by the Editor-in-Chief of this journal on request.

## Sources of funding

No funding was applicable.

## Author contribution

M.H.: concept and design, data interpretation, writing paper, and collecting data; B.T.: concept and design, data analysis and interpretation, and writing paper; M.I., A.Z., A.S.S.A., and I.E.S.: data collection, data interpretation, and writing the paper; M.H.: logistics liraglutide and placebo pens, and writing paper.

## Conflicts of interest disclosure

All authors have no conflicts of interest.

## Research registration unique identifying number (UIN)

It was registered at clinical trials.gov under the NCT number: NCT05285397.

## Guarantor

Mohamed Hany and Bart Torensma.

## Data availability statement

Available upon reasonable request. All data is available with the corresponding author. The analysis was performed on a blinded dataset after the completion of medical/scientific review. All protocol violations were identified and resolved, and the dataset was declared complete. All data were collected in a data management system (Castor EDC, Amsterdam, The Netherlands; https://www.castoredc.com), handled according to Good Clinical Practice guidelines, Data Protection Directive certificate, and complied with Title 21 CFR Part 11. Furthermore, the data centers, where all the research data were stored, were certified according to ISO27001, ISO9001, and Dutch NEN7510.

## Provenance and peer review

Not commissioned, externally peer-reviewed.

## Supplementary Material

SUPPLEMENTARY MATERIAL

## References

[1] Angrisani L Santonicola A Iovino P . Bariatric surgery survey 2018: similarities and disparities among the 5 IFSO chapters. Obes Surg 2021;31:1937–1948. 33432483 10.1007/s11695-020-05207-7 PMC7800839

[2] Guan B Chong TH Peng J . Mid-long-term revisional surgery after sleeve gastrectomy: a systematic review and meta-analysis. Obes Surg 2019;29:1965–1975. 30903425 10.1007/s11695-019-03842-3

[3] Hany M Zidan A Elmongui E . Revisional Roux-en-Y gastric bypass versus revisional one-anastomosis gastric bypass after failed sleeve gastrectomy: a randomized controlled trial. Obes Surg 2022;32:3491–3503. 36098907 10.1007/s11695-022-06266-8 PMC9469810

[4] Pędziwiatr M Małczak P Wierdak M . Revisional gastric bypass is inferior to primary gastric bypass in terms of short- and long-term outcomes—systematic review and meta-analysis. Obes Surg 2018;28:2083–2091. 29748735 10.1007/s11695-018-3300-2 PMC6018598

[5] Hany M Zidan A Sabry K . How good is stratification and prediction model analysis between primary and revisional Roux-en-Y Gastric bypass surgery? A multi-center study and narrative review. Obes Surg 2023;33:1431–1448. 36905504 10.1007/s11695-023-06532-3 PMC10156787

[6] Thakur U Bhansali A Gupta R . Liraglutide augments weight loss after laparoscopic sleeve gastrectomy: a randomised, double-blind, placebo-control study. Obes Surg 2021;31:84–92. 32656729 10.1007/s11695-020-04850-4

[7] Astrup A Rössner S Van Gaal L . Effects of liraglutide in the treatment of obesity: a randomised, double-blind, placebo-controlled study. Lancet 2009;374:1606–1616. 19853906 10.1016/S0140-6736(09)61375-1

[8] Colbourne JRM Fisher OM . The role of adjuvant pharmacotherapy with liraglutide for patients with inadequate weight loss following bariatric surgery. Langenbecks Arch Surg 2023;408:115. 36867261 10.1007/s00423-023-02805-8 PMC9984502

[9] Pajecki D Halpern A Cercato C . Short-term use of liraglutide in the management of patients with weight regain after bariatric surgery. Rev Col Bras Cir 2013;40:191–195. 23912365 10.1590/s0100-69912013000300005

[10] Wharton S Kuk JL Luszczynski M . Liraglutide 3.0 mg for the management of insufficient weight loss or excessive weight regain post‐bariatric surgery. Clin Obes 2019;9:e12323. 31183988 10.1111/cob.12323 PMC6771702

[11] Horber FF Steffen R . Reversal of long-term weight regain after Roux-en-Y gastric bypass using liraglutide or surgical revision. a prospective study. Obes Surg 2021;31:93–100. 32691401 10.1007/s11695-020-04856-y PMC7808975

[12] Elhag W El Ansari W . Effectiveness and safety of liraglutide in managing inadequate weight loss and weight regain after primary and revisional bariatric surgery: anthropometric and cardiometabolic outcomes. Obes Surg 2022;32:1005–1015. 35060021 10.1007/s11695-021-05884-y

[13] Rye P Modi R Cawsey S . Efficacy of high-dose liraglutide as an adjunct for weight loss in patients with prior bariatric surgery. Obes Surg 2018;28:3553–3558. 30022424 10.1007/s11695-018-3393-7

[14] Schulz KF Altman DG Moher D . CONSORT 2010 statement: updated guidelines for reporting parallel group randomised trials. J Pharmacol Pharmacother. 2010;1:100–7. 10.4103/0976-500X.72352 PMC3043330 21350618

[15] El Ansari W Elhag W . Weight regain and insufficient weight loss after bariatric surgery: definitions, prevalence, mechanisms, predictors, prevention and management strategies, and knowledge gaps—a scoping review. Obes Surg 2021;31:1755–1766. 33555451 10.1007/s11695-020-05160-5 PMC8012333

[16] Mehta A Marso SP Neeland IJ . Liraglutide for weight management: a critical review of the evidence. Obes Sci Pract 2017;3:3–14. 28392927 10.1002/osp4.84 PMC5358074

[17] Gero D Vannijvel M Okkema S . Defining global benchmarks in elective secondary bariatric surgery comprising conversional, revisional, and reversal procedures. Ann Surg 2021;274:821–828. 34334637 10.1097/SLA.0000000000005117

[18] Clavien PA Barkun J De Oliveira ML . The Clavien-Dindo classification of surgical complications: five-year experience. Ann Surg 2009;250:187–196. 19638912 10.1097/SLA.0b013e3181b13ca2

[19] Andalib A Alamri H Almuhanna Y . Short-term outcomes of revisional surgery after sleeve gastrectomy: a comparative analysis of re-sleeve, Roux en-Y gastric bypass, duodenal switch (Roux en-Y and single-anastomosis). Surg Endosc 2021;35:4644–4652. 32780238 10.1007/s00464-020-07891-z

[20] Rayman S Assaf D Azran C . Sleeve gastrectomy failure—revision to laparoscopic one-anastomosis gastric bypass or Roux-n-Y gastric bypass: a multicenter study. Obes Surg 2021;31:2927–2934. 33765292 10.1007/s11695-021-05334-9

[21] Ceha CMM Van Wezenbeek MR Versteegden DPA . Matched short-term results of SADI versus gbp after sleeve gastrectomy. OBES SURG 2018;28:3809–3814. 30039236 10.1007/s11695-018-3415-5

[22] Mok J Adeleke MO Brown A . Safety and efficacy of liraglutide, 3.0 mg, once daily vs placebo in patients with poor weight loss following metabolic surgery: the BARI-OPTIMISE randomized clinical trial. JAMA Surg 2023;158:1003. 37494014 10.1001/jamasurg.2023.2930 PMC10372755

[23] Hany M Demerdash HM Zidan A . Effect of weight regain on body composition and metabolic biomarkers after sleeve gastrectomy: a cross-sectional study from a hospital database. Obes Surg 2023;33:268–278. 36462120 10.1007/s11695-022-06384-3 PMC9834094

[24] Perakakis N Kokkinos A Peradze N . Circulating levels of gastrointestinal hormones in response to the most common types of bariatric surgery and predictive value for weight loss over one year: Evidence from two independent trials. Metabolism 2019;101:153997. 31672446 10.1016/j.metabol.2019.153997

[25] Farr OM Tsoukas MA Triantafyllou G . Short-term administration of the GLP-1 analog liraglutide decreases circulating leptin and increases GIP levels and these changes are associated with alterations in CNS responses to food cues: a randomized, placebo-controlled, crossover study. Metabolism 2016;65:945–953. 27282865 10.1016/j.metabol.2016.03.009 PMC4902873

[26] Suliman M Buckley A Al Tikriti A . Routine clinical use of liraglutide 3 mg for the treatment of obesity: Outcomes in non‐surgical and bariatric surgery patients. Diabetes Obes Metab 2019;21:1498–1501. 30768836 10.1111/dom.13672

[27] Yorke E Sheppard C Switzer NJ . Revision of sleeve gastrectomy to Roux-en-Y Gastric Bypass: a Canadian experience. Am J Surg 2017;213:970–974. 28416180 10.1016/j.amjsurg.2017.04.003

[28] Parmar CD Mahawar KK Boyle M . Conversion of sleeve gastrectomy to Roux-en-Y gastric bypass is effective for gastro-oesophageal reflux disease but not for further weight loss. Obes Surg 2017;27:1651–1658. 28063112 10.1007/s11695-017-2542-8

[29] Gero D Raptis DA Vleeschouwers W . Defining global benchmarks in bariatric surgery: a retrospective multicenter analysis of minimally invasive Roux-en-Y gastric bypass and sleeve gastrectomy. Ann Surg 2019;270:859–867. 31592894 10.1097/SLA.0000000000003512

