# Supplementary material for: Boosting weight loss after conversional Roux-en-Y Gastric Bypass with liraglutide and placebo use. A double-blind-randomized controlled trial
Source: Int J Surg. 2023 Dec 14;110(3):1546–55. doi: 10.1097/JS9.0000000000000990 (PMC10942244; doi:10.1097/JS9.0000000000000990)
Supplement: SUPPLEMENTARY MATERIAL [file js9-110-1546-s002.docx]

**Figure 1: CONSORT flow diagram**
