# Supplementary material for: Boosting weight loss after conversional Roux-en-Y Gastric Bypass with liraglutide and placebo use. A double-blind-randomized controlled trial
Source: Int J Surg. 2023 Dec 14;110(3):1546–55. doi: 10.1097/JS9.0000000000000990 (PMC10942244; doi:10.1097/JS9.0000000000000990)
Supplement: SUPPLEMENTARY MATERIAL [file js9-110-1546-s003.docx]

**Appendix 2 Pre- and post-operative Care**

In the outpatient clinic, a multi-disciplinary team assessed all participants, including a bariatric surgeon, a nutritionist, a psychiatrist, and an endocrinologist. The bariatric surgeon explained the procedure with the expected benefits and drawbacks to the participants. The nutritionist explained the nutrition plan and the expected effects of the additional liraglutide use. The endocrinologist assessed the medical condition and associated medical problems of the participants. The psychiatrist identified and supported patients with eating disorders or psychological problems.

Pre-operative upper gastrointestinal endoscopy (UGE) and abdominal ultrasound examination were routinely done for all patients. The presence of a hiatal hernia was assessed by UGE, and GERD was classified according to the Los Angles classification ^1^. Pre-operative laboratory tests included routine tests and a hormonal assay for the pancreas and gut hormones. Enoxaparin was used for prophylaxis against venous thrombotic events (VTE), starting 12 hours before surgery and continuing for at least three weeks after surgery. UGE was performed only for patients with persistent symptoms suspicious of reflux or stomal ulcers, such as epigastric pain, melena, and bilious vomiting. CT imaging was performed for patients with suspicious symptoms and signs of complications such as bleeding, leak, and intestinal obstruction. Oral supplements include multivitamins and minerals such as iron and calcium.

1. Sami S, Ragunath K. The Los Angeles Classification of Gastroesophageal Reflux Disease. *Video Journal and Encyclopedia of GI Endoscopy*. 2013;1:103–104.
