# Supplementary material for: Boosting weight loss after conversional Roux-en-Y Gastric Bypass with liraglutide and placebo use. A double-blind-randomized controlled trial
Source: Int J Surg. 2023 Dec 14;110(3):1546–55. doi: 10.1097/JS9.0000000000000990 (PMC10942244; doi:10.1097/JS9.0000000000000990)
Supplement: SUPPLEMENTARY MATERIAL [file js9-110-1546-s004.docx]

**Appendix 3: Measurement of Metabolic and Hormonal Biomarkers**

Peripheral blood samples were collected and allowed to clot at room temperature for 30 min, followed by centrifugation at 4000 rpm for 10 min at 4 °C. Subsequently; the serum was stored at − 80 °C for analysis. Fasting samples were obtained after overnight fasting for the fasting levels of glucose, insulin, HbA1c, leptin, ghrelin, cholesterol, and triglycerides. Post-prandial samples were collected for GLP-1, PYY, and GIP hormones, immediately and 120 minutes after a standard meal (300 kcal) comprising 20% protein, 35% fat, and 45% carbohydrates ingested within 20 min. pre-operative samples were collected one week before surgery and post-intervention samples were collected after 6 months of the treatment with liraglutide/placebo. All the measurements were analyzed according to standardized operating procedures.

**Hormonal Measurements**

Serum glucose, and lipid profile were measured enzymatically on a Hitachi 7180 Biochemistry Automatic Analyzer (Hitachi, Japan). Fasting insulin levels were measured using ELISA (EIA-2935) [DRG International, Inc. Springfield NJ, USA].

Serum ghrelin was measured using ELISA Kit (Cloud- Clone Corp; cat no: E-01720hu) [W. Fernhurst Dr., Unit 2201, Katy, TX 77,494, USA]). Serum leptin was evaluated by ELISA Kit (Cloud- Clone Corp; cat no: E-00916hu) (TX 77,494, USA). Human Peptide YY measured by ELISA Kit (Cloud- Clone Corp; Cat no: E-01191hu) (TX 77,494, USA).

Homeostasis model assessment of insulin resistance (HOMA-IR) was used to evaluate insulin resistance (fasting serum insulin (μIU/mL) × fasting plasma glucose (mmol/L)/22.5).
