# Supplementary material for: Boosting weight loss after conversional Roux-en-Y Gastric Bypass with liraglutide and placebo use. A double-blind-randomized controlled trial
Source: Int J Surg. 2023 Dec 14;110(3):1546–55. doi: 10.1097/JS9.0000000000000990 (PMC10942244; doi:10.1097/JS9.0000000000000990)
Supplement: SUPPLEMENTARY MATERIAL [file js9-110-1546-s005.docx]

**Appendix 4**

**Surgical technique; the cRYGB Operation**

Standard five ports were used, including three 12-mm ports (for the camera, right and left working ports) and two 5-mm ports (for liver retraction and for the assistant). Pneumo-peritoneum was created after using optical trocars for entry, paying attention to the presence of adhesions from previous surgery. Starting with the dissection of adhesions around the gastric sleeve using the energy device EnSeal® (Ethicon Endo-Surgery, Cincinnati, OH, USA). The gastric pouch was created starting at 5–6 cm below the esophagogastric junction using Echelon Flex Endopath 60-mm linear stapler (Ethicon Endo-Surgery, Cincinnati, OH, USA) over a 40 fr bougie, using gold and blue reloads. The same stapler was used to construct the gastro-jejunostomy and the jejuno-jejunostomy using blue and white reloads, respectively, with equal 100-cm biliopancreatic and alimentary limbs. In two layers, the stapling defects were closed using barbed sutures, 3/0 V-Loc 180 sutures (Covidien, Mansfield, MA, USA). The staple line in the gastric pouch and the remnant stomach were reinforced with seromuscular continuous sutures using the same barbed sutures. All mesenteric defects were closed using 3/0 V-Loc non-absorbable sutures (Covidien, Mansfield, MA, USA).
